# Proteomic Signatures of Adiposomes Track Cardiometabolic Risk Reduction Following Bariatric Surgery

**DOI:** 10.3390/ijms27114939

**Published:** 2026-05-29

**Authors:** Monica C. Asada, Mohamed Saad Rakab, Imaduddin Mirza, Giorgia Scichilone, Mohammed H. Morsy, Amro Mostafa, Francesco M. Bianco, Mohamed M. Ali, Chandra Hassan, Mario A. Masrur, Abeer M. Mahmoud

**Affiliations:** 1 Department of Medicine, Division of Endocrinology, Diabetes, and Metabolism, College of Medicine, University of Illinois Chicago, Chicago, IL 60612, USA; mchen234@uic.edu (M.C.A.); mmirza24@uic.edu (I.M.); gscic@uic.edu (G.S.); mmorsy3@uic.edu (M.H.M.); 2 Faculty of Medicine, Mansoura University, Mansoura 35516, Egypt; mohamedrikab2000@gmail.com; 3 Department of Pharmacology, College of Medicine, University of Illinois Chicago, Chicago, IL 60612, USA; amost2@uic.edu; 4 Department of Surgery, College of Medicine, University of Illinois Chicago, Chicago, IL 60612, USA; biancofm@uic.edu (F.M.B.); chandrar@uic.edu (C.H.); masrur@uic.edu (M.A.M.); 5 Department of Biobehavioral Nursing Science, College of Nursing, University of Illinois Chicago, Chicago, IL 60612, USA; mali37@uic.edu; 6 Department of Kinesiology and Nutrition, College of Applied Health Sciences, University of Illinois Chicago, Chicago, IL 60612, USA

**Keywords:** adiposomes, bariatric surgery, extracellular vesicles, obesity, proteomics, subcutaneous adipose tissue, inflammation, endothelial dysfunction, lipid metabolism, machine learning

## Abstract

Adipose tissue-derived extracellular vesicles (adiposomes) carry a protein cargo that we previously showed differs between obese and lean individuals. In this study, we investigate how adiposomal protein cargo changes in response to sleeve gastrectomy and examine whether these changes are associated with clinical improvements. Twenty-three obese adults underwent pre- and post-bariatric surgery adipose sampling for adiposome isolation and clinical assessments that included vascular and metabolic profiles and inflammatory markers. The adiposomal protein cargo was analyzed via non-targeted proteomics. Differential protein abundance, pathway enrichment, and correlation analyses were assessed. Twelve weeks after bariatric surgery, BMI and fat mass decreased, accompanied by improved glucose and lipid profiles. Inflammatory markers (leptin, IL-6, CRP) also declined, while adiponectin and nitric oxide increased. Adiposomal proteomics identified 287 proteins, with 138 significantly altered. Downregulated proteins included PRDX2, FN1, SERPIND1, and inflammatory mediators; upregulated proteins included talin-1, fibrinogens, and adiponectin. Correlation analysis linked these changes to improvements in lipid profiles, vascular function, and circulating inflammatory markers. Pathway analysis revealed inhibition of lipid-regulatory pathways alongside enrichment of immune, metabolic, and vascular pathways, including lipoprotein metabolism and endothelial signaling. Bariatric surgery-induced cardiometabolic improvements were accompanied by adiposome proteomic remodeling, characterized by reduced inflammation and metabolic reprogramming.

## 1. Introduction

Obesity (BMI ≥ 30 kg/m^2^) affects about 12.5% of the population worldwide [1]. In the United States, over 40% of adults are obese, and it is estimated that this percentage will reach up to 50% by the 2030s [2]. Obesity is considered a public health problem that increases the risk of several health conditions, including metabolic diseases like type 2 diabetes, cardiovascular disorders such as atherosclerosis, musculoskeletal diseases, neuropsychiatric disorders, and various cancers [3]. Therefore, developing effective interventions and investigating the mechanisms by which they modify obesity-associated co-morbidities and underlying molecular mechanisms has become an urgent priority. Obesity is defined by excessive and dysregulated adipose tissue, which was once considered a simple fat reservoir. Nevertheless, it is now recognized as an important excretory organ that plays a role in metabolic regulation, hormone homeostasis, immune response, cognitive health, and more [4]. Adipose tissue releases numerous bioactive molecules, and a significant portion of this secretome can be found in adipocyte-derived extracellular vesicles, referred to as AdEVs or adiposomes, as we previously described [5,6,7].

Adiposomes are lipid envelopes that carry genetic materials, proteins, lipids, and other metabolites. These vesicles act as important inter-organ communicators that are capable of incorporating their cargo into other cells and tissues in a paracrine manner or remotely [8]. We and others have demonstrated that the number of adiposomes increases in dysregulated adipose tissue in obesity [9,10]. Using adipose tissue biopsies, our prior studies established the molecular cargo of adiposomes as mechanistic mediators of vascular dysfunction and predictive biomarkers of inflammation, insulin resistance, and metabolic disease [9,11,12,13]. In a cohort of 122 individuals, lipidomics profiling of Ad-adiposomes showed enrichment in ceramides, FFAs, acylcarnitines, and inflammatory lipids, and depletion of vasoprotective lipid species like FAHFAs and phosphocholines in obese participants compared to lean controls [9,14]. These patterns correlated with insulin resistance, systemic inflammation, impaired NO bioavailability, and reduced arteriolar vasodilation. Proteomic analyses further identified enrichment of inflammatory proteins, such as CRP and C9, and loss of protective proteins, such as adiponectin, with pathway enrichment analysis highlighting TNFα/IL-1–driven pathways. These omics profiles predicted T2D and hypertension in the obese cohort with >85% accuracy, supporting their translational potential as biomarkers of cardiometabolic diseases [13]. However, whether these adiposomal proteomic signatures are dynamically remodeled in response to interventions that produce substantial fat-mass reduction, such as bariatric surgery, remains unknown.

Bariatric surgery is considered one of the most effective therapies, especially for patients with severe obesity (BMI ≥ 40 kg/m^2^) or associated comorbidities. Previous studies showed more than 20% loss of body weight after bariatric surgery within the first two years, accompanied by sustained improvements in metabolic indicators such as HbA1c and triglycerides [15]. Bariatric surgery ameliorates common comorbidities of obesity, including hypertension, hyperlipidemia, insulin resistance, type II diabetes, hepatic steatosis, cardiovascular diseases, and systemic inflammation, while also lowering the risk of all-cause mortality [16,17,18]. In our studies, we observed reductions in the adiposome cargo of inflammatory and lipotoxic lipids (e.g., ceramides, acylcarnitine, and triglycerides), along with increases in potentially vasculoprotective lipid cargo such as esterified fatty acids (FAHFAs), following bariatric surgery [19]. These changes mirrored post-bariatric improvements in BMI, vasoreactivity, insulin sensitivity, and systemic inflammation. Building on these findings, the current study investigates longitudinal changes in adiposomal protein cargo in the same individuals before and after bariatric surgery and examines whether these proteomic shifts are associated with improvements in cardiometabolic health.

## 2. Results

### 2.1. Clinical Characteristics of the Study Participants

Demographic and comorbidity information of the participants is summarized in Table 1. At baseline, the bariatric surgery cohort was composed of 23 obese adults, predominantly female (87%), with a mean age of 36.7 ± 7.74 years. The racial distribution was mainly African American (52%), followed by Hispanic (39%) and non-Hispanic White (9%). As expected for a population eligible for bariatric surgery, the burden of cardiometabolic comorbidities was high; among them, 52% were hypertensive, 39% diabetic, 65% insulin resistant, and 65% had dyslipidemia. Notably, hepatic steatosis was present in 74% of participants, and vascular impairment was nearly universal; 91% had impaired flow-mediated dilation (FMD), 100% had impaired flow-induced dilation (FID), and 70% showed evidence of impaired vascular function. Systemic inflammation was also common, affecting 61% of the cohort. 

### 2.2. Post-Surgical Cardiometabolic Improvements

**Figure 1:**
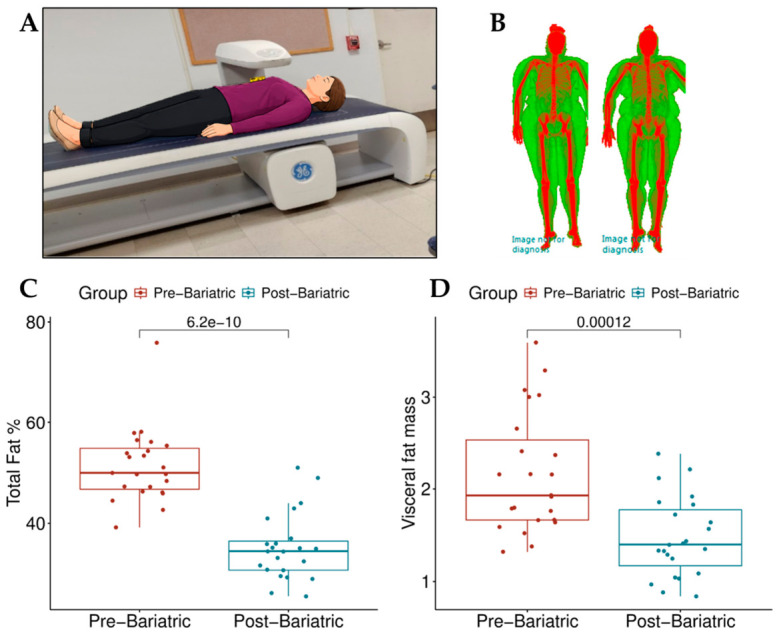
Anthropometric improvements after bariatric surgery. (**A**,**B**) Representative images of DEXA scanning. (**C**,**D**) Pre- and post-bariatric measurements of total fat percentage and visceral fat mass. Pre- versus post-surgery comparisons were paired and analyzed using the paired Student’s *t*-test for normally distributed variables or the Wilcoxon signed-rank test for non-normally distributed variables, as determined by the Shapiro–Wilk test. Data are shown as mean ± SD.

Three months after undergoing bariatric surgery, participants exhibited significant reductions in body weight and fat mass. Mean BMI decreased by 9.7 ± 5.45 kg/m^2^ (*p* < 0.01), and both total fat percent and visceral fat mass (measured via DEXA scanning) were remarkably reduced (*p* < 0.01; Figure 1).

**Figure 2:**
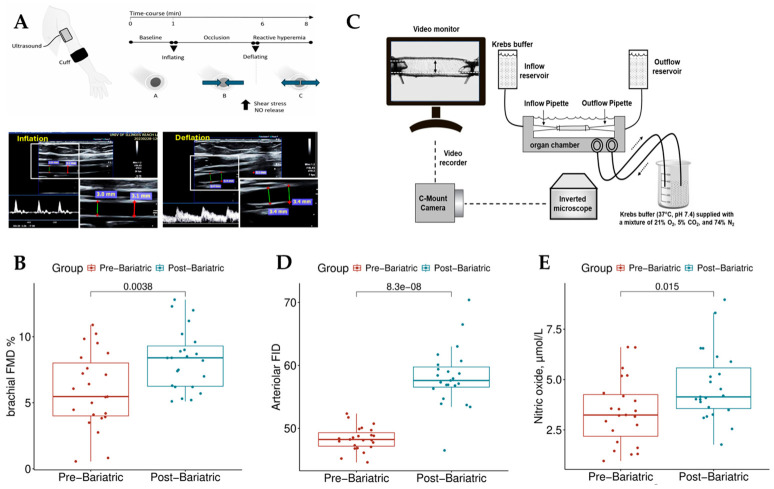
Vascular improvements after bariatric surgery. (**A**) Representative images of brachial artery ultrasound imaging for brachial flow-mediated dilation (FMD). (**B**) Box plot for pre- and post-bariatric brachial FMD%. (**C**) A schematic illustration of the microvascular measurement setup for measuring flow-induced dilation (FID) of adipose tissue-isolated arterioles. (**B**,**D**) Box plot for pre- and post-bariatric arteriolar FID. (**E**) Box plot for pre- and post-bariatric circulating nitric oxide. Pre- versus post-surgery comparisons were paired and analyzed using the paired Student’s *t*-test for normally distributed variables or the Wilcoxon signed-rank test for non-normally distributed variables, as determined by the Shapiro–Wilk test.

Resting heart rate declined by approximately 9 ± 15 bpm (*p* < 0.01), and systolic blood pressure improved significantly (−15 ± 19 mmHg, *p* < 0.01) while diastolic blood pressure did not show significant changes (−2 ± 14 mmHg, *p* = 0.4506). We also observed significant improvements in markers of vascular function as evidenced by marked increases in brachial flow-mediated dilation (FMD) (+2.34 ± 4.01%, *p* = 0.0038; Figure 2A,B) and arteriolar flow-induced dilation (FID) measured at Δ60 H_2_O pressure gradient (+9.77 ± 5.38%, *p* < 0.001; Figure 2C,D), suggesting recovery of vascular reactivity in both macro- and microvessels. These findings were accompanied by inductions in nitric oxide (NO) levels (+1.27 ± 2.49 µmol/L; *p* = 0.015; Figure 2E), which may contribute to the observed vascular improvements.

Significant improvements were observed in metabolic biomarkers as well (Table 2). Fasting insulin and fasting glucose levels decreased by 5.86 ± 7.32 µIU/mL and 24.22 ± 9.93 mg/dL, respectively (*p* < 0.01). Homeostasis model assessment of insulin resistance (HOMA-IR), calculated as fasting glucose (mg/dL) x fasting insulin (µIU/mL)/405, was also reduced (−2.46 ± 3.14; *p* < 0.01), reflecting improved insulin sensitivity. Even though post-surgical fasting insulin, fasting glucose, and HOMA-IR remained within the normal range (<10 mg/dL, 70–90 µIU/mL, and <2.0–2.5, respectively), improvements observed were significant. At the same time, HbA1c (hemoglobin A1c, or glycosylated hemoglobin), a standard prognostic tool for prediabetes and type 2 diabetes, dropped by 1.25 ± 1.52% (*p* < 0.01), consistent with improved glycemic control. Lipid profiles also improved: triglyceride levels declined by 37.78 ± 80.58 mg/dL (*p* = 0.0355); total cholesterol was reduced by 33.43 ± 53.92 mg/dL (*p* < 0.01), LDL decreased by 18.04 ± 39.46 mg/dL (*p* = 0.0392), and HDL increased by 8.74 ± 17.01 mg/dL (*p* = 0.0220). Liver-related markers exhibited variable responses following bariatric surgery. Alkaline phosphatase (ALP) levels decreased significantly by 16.00 ± 21.32 U/L (*p* < 0.01), indicating a potential improvement in hepatobiliary function or reduced hepatic stress, while changes in AST (−4.35 ± 24.81 U/L, *p* = 0.4098) and ALT (−4.96 ± 38.87 U/L, *p* = 0.5471) were not significant, suggesting that early postoperative effects on hepatocellular injury may be rather modest. Total bilirubin levels decreased slightly (−0.09 ± 0.27 mg/dL, *p* = 0.1228), which was not statistically significant, but remained aligned with overall trends toward liver function normalization. Total protein decreased by 1.47 ± 0.99 g/dL (*p* < 0.01) while serum albumin levels also declined (−0.87 ± 0.56 g/dL, *p* < 0.01). Conversely, hemoglobin increased significantly by 2.52 ± 2.21 g/dL (*p* < 0.01), likely indicating improved erythropoietic status post-surgery (Table 2). Changes in anthropometric, metabolic, vascular, and inflammatory parameters before and after bariatric surgery (n = 23).

**Figure 3:**
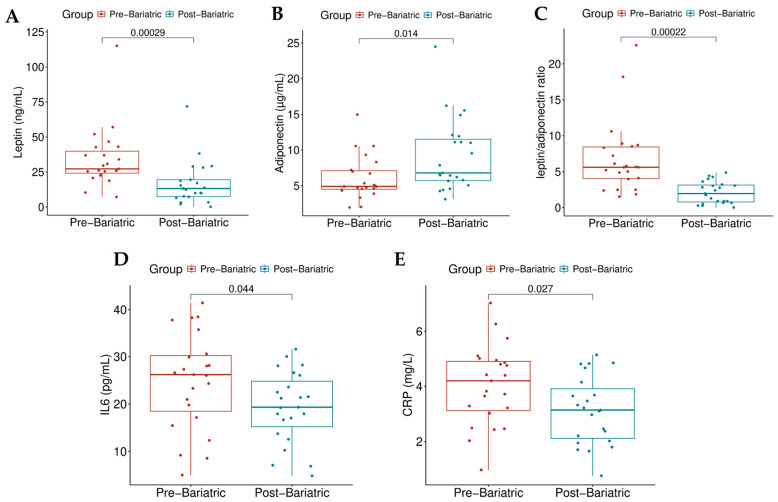
Changes in circulating adipokines after bariatric surgery. Box plots for pre- and post-bariatric levels of circulating leptin (**A**), adiponectin (**B**), leptin/adiponectin ratio (**C**), IL6 (**D**), and CRP (**E**). Pre- versus post-surgery comparisons were paired and analyzed using the paired Student’s *t*-test for normally distributed variables or the Wilcoxon signed-rank test for non-normally distributed variables, as determined by the Shapiro–Wilk test.

Adipokines such as leptin declined sharply (−17.84 ± 13.53 ng/mL, *p* < 0.001; Figure 3A), while adiponectin increased (+2.64 ± 4.40 µg/mL, *p* = 0.014; Figure 3B), resulting in a significant reduction in the leptin/adiponectin ratio (−1.49 ± 3.72, *p* < 0.001; Figure 3C), a vital indicator of metabolic health. Finally, we observed marked reductions in the inflammatory markers, including IL-6 (−5.49 ± 10.68 pg/mL, *p* = 0.044; Figure 3D) and CRP (−0.90 ± 2.05 mg/L, *p* = 0.027; Figure 3E), consistent with reduced systemic inflammation following weight loss. Together, these changes highlight rapid improvements in vascular and metabolic function, as well as systemic inflammation, within 3 months after bariatric surgery.

### 2.3. Post-Bariatric-Surgery Proteomics Shifts

**Figure 4:**
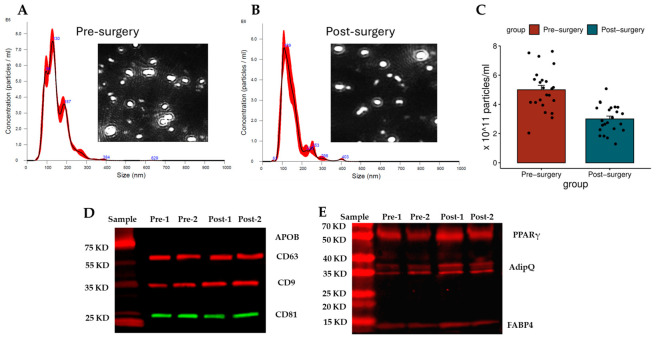
Characterization of adiposomes from human SAT before and after bariatric surgery. (**A**,**B**) Nanoparticle tracking analysis (NTA) shows the size distribution and concentration of adiposomes isolated from SAT collected pre- and post-surgery, with vesicles primarily in the small EV range (~50–300 nm). The insert is a representative image of the NTA-detected particles. (**C**) Quantification of particle concentration shows a reduction in circulating adiposomes after surgery. (**D**,**E**) Western blots demonstrate the presence of established EV markers and adipocyte-specific proteins. In (**C**), pre- versus post-surgery particle concentrations were compared by paired Student’s *t*-test or Wilcoxon signed-rank test, as appropriate based on Shapiro–Wilk normality testing.

To study adiposome lipidomics, we isolated adiposomes from subcutaneous adipose tissue (SAT) biopsies obtained from participants before and after bariatric surgery. Adiposomes were quantified using nanoparticle-tracking analysis (NTA). Their sizes ranged from 50 to 300 nm, and their numbers were significantly lower after surgery compared to before surgery (6.3 × 10^11^ particles/mL vs. 8.1 × 10^11^ particles/mL, *p* < 0.001) (Figure 4A–C). Adiposome vesicular and adipocytic nature was confirmed via Western blotting using specific antibodies for tetraspanins (CD9, CD81, and CD63) and adipocytic proteins (PPARγ, adiponectin, and fatty acid-binding protein 4 [FABP4]). There was also no detectable contamination from lipoproteins (Apolipoprotein B; APOB) (Figure 4D,E).

**Figure 5:**
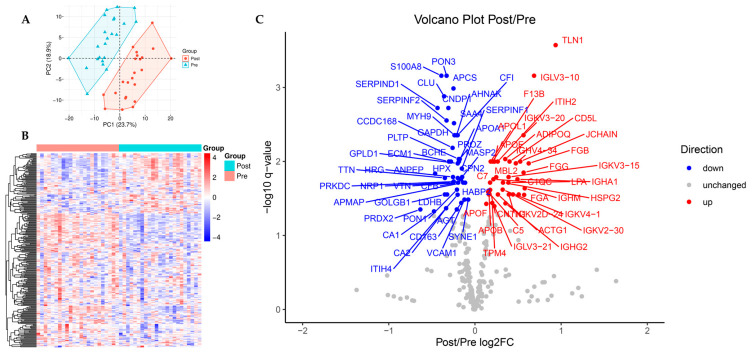
Differential adiposome proteins in post- and pre-surgery individuals. (**A**) Principal Component Analysis (PCA) plot of adiposome proteins in post- and pre-surgery groups. (**B**) Heatmap of significantly different adiposome proteins in post- and pre-surgery groups. (**C**) Volcano plot of differential protein expression (log_2_FC) between groups (-log_10_ *q*-value). Differential abundance was assessed on paired pre- and post-surgery samples using paired Student’s *t*-test or Wilcoxon signed-rank test as appropriate, with multiple-testing correction by the Benjamini–Hochberg false discovery rate (FDR); proteins with FDR-adjusted *q* < 0.05 are highlighted.

To assess short-term changes in the proteomic composition of adiposomes following bariatric surgery, a differential abundance analysis was performed on paired samples collected from the same individuals prior to and 12–14 weeks post-surgery. Unbiased quantitative proteomic analysis identified 282 unique proteins. Subsequent downstream differential abundance analysis found 138 proteins that exhibited statistically significant changes (FDR < 0.05; log_2_FC ≠ 0). To confirm that key findings were not driven by the choice of FDR threshold, the differential abundance analysis was re-run at a stricter 1% protein-level FDR; the direction and identity of the top significantly altered proteins (including TLN1, PRDX2, FN1, SERPIND1, CRP, S100A8, FGA/FGB/FGG, and the immunoglobulin set) were preserved, indicating that the proteomic signature reported here is robust to the FDR cutoff. Based on this differential analysis, the principal component analysis (PCA) plot revealed a clear separation between pre- and post-surgery samples, with PC1 and PC2 accounting for 45.5% of the total variance (24.9% and 20.6%, respectively), indicating consistent proteomic alterations following bariatric surgery (Figure 5A). Coherent changes in protein abundance and group-specific trends were also evident in the heatmap (Figure 5B).

**Figure 6:**
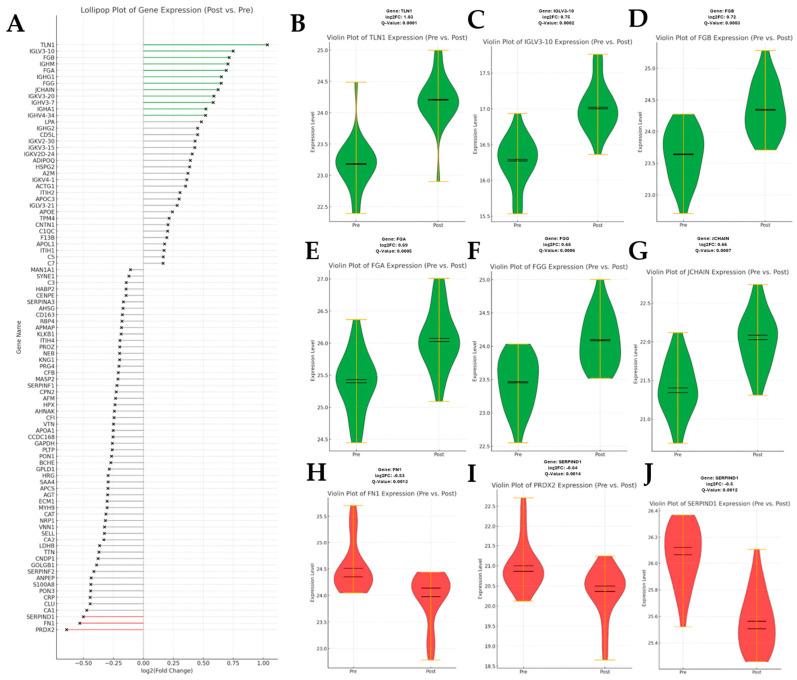
Differential protein profiles in adiposomes of post- and pre-surgery groups. (**A**) Lollipop plot of proteins with statistically significant changes stratified by biological significance. Red: upregulated (log_2_FC > 0.5, *q* < 0.05); green: downregulated (log_2_FC < −0.5, *q* < 0.05); gray: not biologically significant. (**B**–**J**) Violin plots showing examples of the significantly altered proteins between the post- and pre-surgery groups. Differential abundance was assessed on paired pre- and post-surgery samples by paired Student’s *t*-test or Wilcoxon signed-rank test as appropriate, with multiple-testing correction by the Benjamini–Hochberg false discovery rate (*q*-values).

Protein-level changes and statistical significance were visualized in the volcano plot, which illustrated the distribution of differentially abundant proteins (Figure 5C). To further highlight the direction and extent of changes, a lollipop plot was generated to show the significantly altered proteins, ranked from the most upregulated to the most downregulated (|log_2_FC| > 0.5/FC > 1.4, *q*-value < 0.05) in the post-surgery group (Figure 6A).

Among the upregulated proteins, the most increased was talin-1 (TLN1), with a log_2_FC of 0.5 (*q* = 0.0001) (Figure 6B). TLN1 is a cytoskeletal protein that links integrins to the actin cytoskeleton and plays a critical role in cell adhesion and migration, suggesting enhanced cellular remodeling post-surgery. Multiple immunoglobulins, including Immunoglobulin Lambda Variable 3-10 (IGLV3-10; Figure 6C), Immunoglobulin Heavy Constant Mu (IGHM), Immunoglobulin Kappa Variable 3-20 (IGKV3-20), Immunoglobulin Heavy Variable 3-7 (IGHV3-7), Immunoglobulin Heavy Constant Alpha 1 (IGHA1), and Immunoglobulin Heavy Variable 4-34 (IGHV4-34), as well as the joining chain of multimeric IgA and IgM (JCHAIN; Figure 6G), increased significantly after bariatric surgery. Other immunoglobulins also reached statistical significance, though with log_2_FC values below 0.5, including Immunoglobulin Heavy Constant Gamma 2 (IGHG2), Immunoglobulin Kappa Variable 2-30 (IGKV2-30), Immunoglobulin Kappa Variable 3-15 (IGKV3-15), Immunoglobulin Kappa Variable 2D-24 (IGKV2D-24), Immunoglobulin Kappa Variable 4-1 (IGKV4-1), and Immunoglobulin Lambda Variable 3-21 (IGLV3-21). This broad increase in immunoglobulin levels may indicate enhanced B-cell activity or improved humoral immune surveillance.

Another group of proteins that increased after surgery was fibrinogens; fibrinogen beta chain (FGB; log_2_FC = −0.72, *q* = 0.003; Figure 6D), fibrinogen alpha chain (FGA; log_2_FC = −0.69, *q* = 0.002; Figure 6E), and fibrinogen gamma chain (FGG; log_2_FC = −0.65, *q* = 0.003; Figure 6F), pointing to restoration of hemostatic function. Interestingly, lipoprotein(a) (LPA), CD5-like molecule (CD5L, also known as apoptosis inhibitor of macrophage, AIM), and adiponectin (ADIPOQ) also increased significantly after surgery (log_2_FC = 0.48, 0.45, 0.39, respectively), indicating an amelioration of metabolic and immune disturbances.

Among the downregulated proteins, peroxiredoxin-2 (PRDX2), a crucial enzyme that protects cells from reactive oxygen species-induced damage, showed the greatest magnitude of reduction (log_2_FC = −0.64; *q* = 0.043), suggesting a potential decrease in oxidative stress (Figure 6I). Fibronectin-1 (FN1) also showed a moderate decrease (log_2_FC = −0.53; *q* = 0.031), which may reflect remodeling of the extracellular matrix or reduced tissue fibrosis (Figure 6H). Serpin family D member 1 (SERPIND1), also known as heparin cofactor 2, decreased by log_2_FC = −0.5 (*q* = 0.0002), implying possible alterations in coagulation balance (Figure 6J). Notably, inflammatory factors such as C-reactive protein (CRP), S100 calcium-binding protein A8 (S100A8), and serum amyloid A4 (SAA4) consistently decreased (log_2_FC = −0.44, −0.44, −0.3; *q* = 0.0336, 0.0002, 0.0005, respectively), suggesting an alleviation of systemic inflammation, which aligns with the circulatory data showing significant reductions in plasma IL6 and CRP levels.

### 2.4. Correlation Between Proteomic Shift and Clinical Measurements

Pearson correlation analysis was performed to assess correlations between changes in protein abundance and changes in clinical data in order to further elucidate the potential clinical relevance of adiposome proteome remodeling in obesity following bariatric surgery. Given the limited sample size (n = 23), correlation magnitudes are described using conventional descriptors (|r| < 0.3 weak, 0.3–0.5 moderate, > 0.5 moderate-to-strong) and are reported with false discovery rate (FDR)-adjusted *p*-values to account for multiple comparisons; these associations should be considered hypothesis-generating rather than confirmatory. The correlation map (Supplementary Figure S1) revealed that the adiposome proteome is related to adiposity, lipid metabolism, glycemic control, inflammation, and cardiovascular function, indicating that the adiposome content is closely tied to systemic cardiometabolic status.

Several proteins showed correlation patterns broadly consistent with their direction of change after bariatric surgery. Among proteins that were decreased after surgery, neuropilin-1 (NRP1), a transmembrane co-receptor involved in angiogenesis and metabolism (e.g., fatty acid transport), was correlated with fasting glucose (r = 0.498, *p* = 0.01558) and HOMA-IR (r = 0.4486, *p* = 0.0317), suggesting that higher adiposome-associated NRP1 tracked with poorer glycemic control. Paraoxonase 3 (PON3), an antioxidant that also plays a role in lipoprotein regulation, was also reduced after surgery and associated with fat percent (r = 0.471, *p* = 0.0232) and visceral fat mass (r = 0.471, *p* = 0.0232), supporting its relationship with obesity and atherosclerosis. Likewise, the carbonic anhydrase isoforms CA1 and CA2, which regulate CO_2_ (bicarbonate) balance and have been associated with metabolic and vascular homeostasis, showed correlations with lipid-related parameters. CA1 correlated with triglycerides (r = 0.583, *p* = 0.0034), total cholesterol (r = 0.507, *p* = 0.0135), and LDL (r = 0.481, *p* = 0.02), whereas CA2 was correlated with LDL (r = 0.6, *p* = 0.0024), total cholesterol (r = 0.597, *p* = 0.0026), and nitric oxide (r = −0.5341, *p* = 0.008656), suggesting a link between this protein subset and dyslipidemia-associated vascular dysfunction. Peroxiredoxin-2 (PRDX2), a redox antioxidant involved in oxidative stress responses as well as the most significantly decreased protein after surgery, showed correlations with triglycerides (r = 0.543, *p* = 0.0073), total cholesterol (r = 0.56, *p* = 0.0054), and LDL (r = 0.518, *p* = 0.0112). Together, these findings suggest that changes in this protein subset aligned with the magnitude of improvement in cardiometabolic parameters after weight loss. On the other hand, several proteins that were increased after surgery showed associations with the magnitude of improvement in metabolic parameters. Contactin-1 (CNTN1), a cell adhesive molecule that has been implicated in the neuro-metabolic axis, was associated with an alteration in IL-6 post-surgically, implying the possibility that its enrichment is linked to ameliorated inflammation during metabolic recovery.

However, some proteins showed strong clinical correlation but did not exhibit clear differential modification after surgery. For example, collagen type VI alpha 3 (COL6A3), an extracellular matrix remodeling protein enriched in fibrosis, was associated with body mass index (r = 0.6554, *p* = 0.000687) and IL-6 (r = 0.4639, *p* = 0.025766), suggesting an association between extracellular matrix dysregulation and inflammatory burden, although it only showed a little appreciable post-surgical change. Likewise, aldolase A (ALDOA), a glycolytic enzyme reflecting cellular metabolic activity, showed strong correlations with leptin (r = 0.585, *p* = 0.0033), the leptin/adiponectin ratio (r = 0.53, *p* = 0.0082), and nitric oxide (r = 0.502, *p* = 0.0145), yet without a clear mean-level post-surgical shift. Complement factor H-related 2 (CFHR2), a regulator of complement activity, demonstrated a similar pattern, correlating with leptin (r = 0.691, *p* = 0.0002) and the leptin/adiponectin ratio (r = 0.77, *p* = 0.000017), yet without clear post-surgical alteration despite having some of the highest correlation scores.

### 2.5. Upstream Regulators and Canonical Pathways Associated with Adiposome Proteomic Shift

To generate hypotheses regarding regulatory mechanisms that may underlie obesity-associated alterations in the adiposome proteome after bariatric surgery, in silico upstream regulator mapping and pathway enrichment analysis were performed using Ingenuity Pathway Analysis. The regulators and pathways reported here are computational predictions inferred from the abundance and direction of change in differentially abundant proteins and were not validated experimentally in this study; they should therefore be regarded as hypothesis-generating. The z-score heatmap (Supplementary Figure S2) visualizes the activation or inhibition of predicted upstream regulators, filtered by *p*-value < 0.05 and |z| > 1.96, inferred from the abundance of their downstream protein targets. Upstream regulator analysis revealed robust inhibition of inflammatory mediators, including INTERLEUKIN-1 BETA (IL1B) (z = −3.00; *p* < 0.005), tumor necrosis factor (TNF) (z = −2.7; *p* < 0.005), IL20 (z = −2.23; *p* < 0.005), and IL6 (z = −1.60; *p* < 0.005), which modulated multiple acute-phase and immune-related targets such as complement component 3 (C3), C5, CRP, and S100A8, suggesting a resolve of pro-inflammation status.

Simultaneously, the nuclear factor kappa-B (NFκB) complex (z = −2.21; *p* < 0.005) was also suppressed, supporting sustained reprogramming of immune and stress responses. Other immunomodulatory cytokines, IL2 (z = −2.375; *p* < 0.005) and IL17A (z = −3.09; *p* < 0.005), were also inhibited, indicating an orchestrated regulation of both innate and adaptive immunity. In contrast, the most significantly activated upstream regulators, including retinoic acid receptor alpha (RARA) (z = 2.22; *p* < 0.005). Collectively, these analyses suggest changes in predicted inflammatory and transcriptional regulatory networks following bariatric surgery, although these inferences require further experimental validation. 

The canonical pathway analysis (Supplementary Figure S3) evaluated the significance and directional shift in biological pathways, incorporating both statistical enrichment (i.e., Benjamini–Hochberg-adjusted (B-H) *p*-values) and activation z-scores to highlight pathways with coherent molecular engagement. The analysis identified liver X receptor/retinoid X receptor (LXR/RXR) (z = −2.4; *p* < 0.005), 24-dehydrocholesterol reductase (DHCR24, also known as seladin-1) (z = −2.52; *p* < 0.005), and inhibitor of DNA binding 3 (ID3) (z = −3.74; *p* < 0.005) (z = −2.83; *p* < 0.005) as significantly inhibited pathways. On the contrary, multiple pathways related to innate immunity, vascular interaction, and coregulatory function showed upregulation after surgery; these included the complement cascade, complement system, binding and uptake of ligands by scavenger receptors, cell–surface interactions at the vascular wall, coagulation system, and Fc gamma receptor (FCGR) dependent phagocytosis. Functionally, these pathways participate in innate immune surveillance, opsonization and phagocytic clearance, lipid and damage-associated ligand uptake, interactivity between immune cells and endothelium, and coagulation-related processes. The postsurgical enrichment of these signaling may reflect enhanced immune recognition and clearance, more efficient handling of circulating hazards (e.g., modified low-density lipoproteins, apoptotic cell debris, and pathogens), and restoration of vascular homeostasis.

Other pathways that were enriched included those related to metabolic functions such as plasma protein assembly and clearance and insulin signaling (e.g., IGF transport via IGFBPs and p70S6K signaling). In addition, enrichment of several vascular and endothelial-related pathways was also observed, including atherosclerosis signaling, production of nitric oxide and reactive oxygen species in macrophages, cell–surface interactions at the vascular wall, and integrin signaling. Moreover, there were also enrichments of coagulation-related pathways, including the coagulation system, prothrombin activation, and fibrin clot formation. Collectively, these enrichment analyses indicate that bariatric surgery is associated with coordinated adiposome-mediated changes in lipid metabolism and vascular signaling, consistent with improved cardiometabolic and endothelial function. Together with the upstream regulator analysis (summarized in Table 3), these results indicate coordinated changes in immune-related pathways following bariatric surgery and suggest potential shifts in immune regulatory processes associated with postoperative metabolic improvement.

### 2.6. Adiposome Protein Signatures as Predictors of Cardiometabolic Risk

To explore, in a hypothesis-generating manner, candidate adiposome protein predictors of post-bariatric changes in BMI, total fat percentage, insulin resistance (HOMA-IR), and brachial artery flow-mediated dilation (FMD), we developed a neural network model that used changes in adiposome proteomic profiles as input variables. Because the cohort was small (n = 23) relative to the high feature dimensionality of the proteomic input space, the analysis is intentionally exploratory, and the metrics reported below should be interpreted with caution; even with 10-fold cross-validation, low error metrics in this setting may, in part, reflect overfitting to the training samples and require external validation in independent cohorts. The model approximated changes in BMI within the training/cross-validation folds, with a testing sum of squares error (SSE) of 0.047 and a relative error of 0.087. Top proteins that contributed to the model fit for BMI changes included neuropilin-1 (NRP1), extracellular matrix protein 1 (ECM1), centromere-associated protein E (CENPE), and inter-alpha-trypsin inhibitor heavy chain 2 (ITIH2). Others with modest contribution included complement component 5 (C5), S100 calcium-binding protein A8 (S100A8), and serpin family D member 1 (SERPIND1). Changes in the proteomics profiles in adiposomes were also associated with post-bariatric changes in fat percentage with moderate accuracy (testing SSE = 0.220, relative error = 0.180), with the topmost predictors to include glycosylphosphatidylinositol-specific phospholipase D1 (GPLD1), immunoglobulin heavy constant alpha 1 (IGHA1), carnosine dipeptidase 1 (CNDP1), and immunoglobulin lambda variable 3-21 (IGLV3-21).

**Figure 7:**
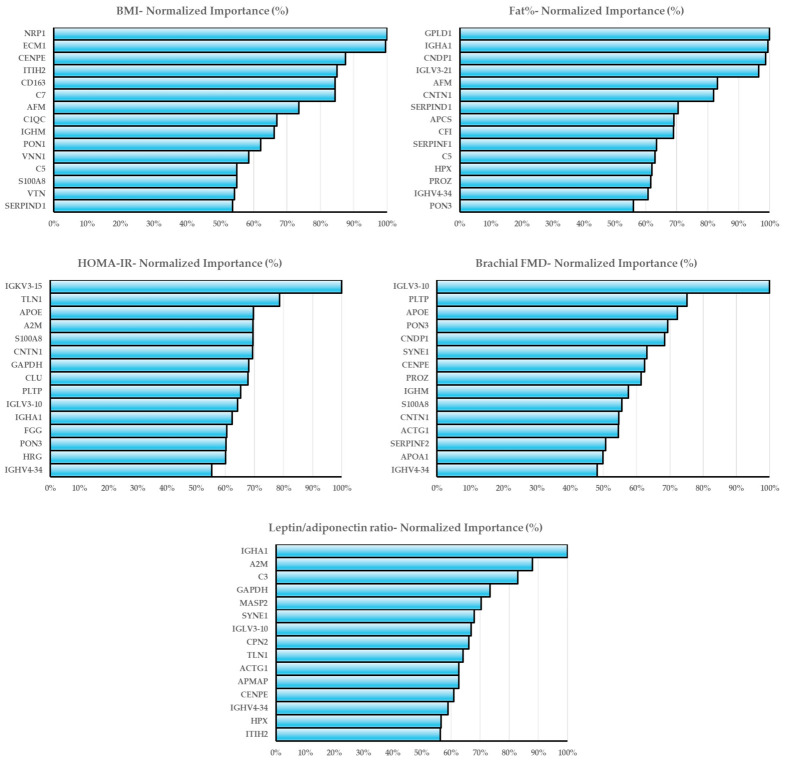
Neural network-derived importance of adiposome proteins predicting clinical outcomes after bariatric surgery. Bar plots show the normalized importance of the top adiposome proteins contributing to neural network models predicting BMI, fat percentage, HOMA-IR, brachial artery flow-mediated dilation (FMD), and the leptin/adiponectin ratio. Importance values were normalized to the top predictor within each model (100%). Multilayer perceptron models were trained in IBM SPSS (v29) using a 70/30 train-test split with 10-fold cross-validation; performance was evaluated by sum of squares error (SSE), R^2^, and mean absolute error (MAE). Given the small cohort (n = 23) relative to feature dimensionality, results are exploratory and require external validation in independent cohorts.

For the insulin resistance index, HOMA-IR, changes in adiposome proteomics were highly predictive, with testing SSE = 0.181 and relative error = 0.30. The top contributors were immunoglobulin kappa variable 3-15 (IGKV3-15), talin-1 (TLN1), apolipoprotein E (APOE), and S100 calcium-binding protein A8 (S100A8). Similarly, brachial artery flow-mediated dilation (FMD) was accurately predicted (testing SSE = 0.059, relative error = 0.102), with top predictors including immunoglobulin lambda variable 3-10 (IGLV3-10), phospholipid transfer protein (PLTP), apolipoprotein E (APOE), and paraoxonase 3 (PON3). Finally, we tested the predictability of this model to the leptin/adiponectin ratio, which showed good predictive performance (testing SSE = 0.648, relative error = 0.20), and the top protein predictors included immunoglobulin heavy constant alpha 1 (IGHA1), alpha-2-macroglobulin (A2M), complement component 3 (C3), and glyceraldehyde-3-phosphate dehydrogenase (GAPDH). Together, our findings suggest that changes in adiosome proteomics observed 3 months after bariatric surgery were informative enough to predict changes in other clinical and physiological parameters, such as adiposity, vascular function, and metabolic regulation. Figure 7 shows the top 15 predictors of post-bariatric changes in BMI, fat percentage, HOMA-IR, brachial artery FMD, and circulating leptin/adiponectin ratio.

## 3. Discussion

The current study demonstrated that the substantial weight loss following bariatric surgery was associated with significant improvements in metabolic and vascular health within three months. Patients showed reductions in BMI, visceral fat, blood pressure, and resting heart rate, along with improved insulin sensitivity and endothelial function. These clinical changes were accompanied by decreased systemic inflammation (CRP, IL-6) and favorable adipokine remodeling, including reduced leptin and increased adiponectin levels, reflecting improved adipose tissue function and metabolic homeostasis.

A central novelty of this study is the proteomic analysis of adiposomes, which revealed extensive molecular changes after weight loss. Of the 287 adiposome proteins identified, 138 (~48%) were significantly regulated post-surgery, highlighting weight loss as a powerful stimulus for altering the adipose tissue milieu and subsequently the content of adipose tissue-derived extracellular vesicles (adiposomes). Strikingly, the direction of change in adiposome proteins was not random, but instead clustered around biological processes central to a shift from a pro-inflammatory, stress-related cargo toward a profile indicative of immune resolution redox balance, lipid handling, extracellular matrix remodeling, and vascular and metabolic homeostasis.

In our previous obese vs. lean adiposome proteomics study, obesity was characterized by enrichment of inflammatory cargo, such as C-reactive protein (CRP) and complement 9 (C9), together with depletion of protective proteins, such as adiponectin, thereby linking adiposome composition to chronic inflammation and cardiometabolic risk [13]. The current longitudinal data extend the findings by showing that adiposome cargo is not static; rather, it is dynamically reprogrammed after bariatric surgery in a direction largely opposite to the obese phenotype. Classical inflammatory markers were among the most downregulated proteins; CRP and SAA4 (acute-phase reactants linked to systemic inflammation), S100A8 (a calgranulin associated with neutrophil/macrophage inflammation and insulin resistance), SERPIND1 (heparin cofactor II, an acute-phase anticoagulant protein), and PRDX2 (peroxiredoxin-2, an antioxidant enzyme often upregulated under oxidative stress) all significantly decreased in adiposomes after surgery. The reduction in these factors was associated with lower levels of the systemic inflammatory markers, IL6 and CRP, observed after surgery. Elevated SAA and S100A8/S100A9 levels are hallmarks of obesity and metabolic syndrome, and weight loss is known to reduce their expression in both adipose tissue and circulation [20]. Our findings extend these observations by showing that, following weight loss, these pro-inflammatory proteins are present at lower levels in adiposomes, potentially limiting their transport to distant organs.

Intriguingly, several overlapping proteins identified in both of our datasets (obese vs. lean healthy and pre- vs. post-bariatric), including PRDX2, SERPIND1, and TLN1, remain relatively underexplored in the context of obesity-associated diseases. The increased abundance of PRDX2 and SERPIND1 in obese individuals and in the pre-bariatric state may reflect adaptive responses to the oxidative and thrombo-inflammatory stress that characterizes severe obesity. PRDX2 is a key antioxidant enzyme involved in peroxide detoxification and redox homeostasis, and its elevation is biologically consistent with the heightened oxidative stress observed in obesity [21]; similarly, oxidative stress is broadly reduced after bariatric surgery, supporting the idea that PRDX2 enrichment may mark the pre-surgical metabolically stressed state [22]. On the other hand, SERPIND1 (also called heparin cofactor II), a thrombin-inhibitory serpin, may represent a compensatory protective response against obesity-associated coagulation imbalance and metabolic inflammation. Prior work has linked SERPIND1 to improved insulin sensitivity and reduced adipose inflammation, suggesting that its elevation in obesity/pre-bariatric state may not simply indicate coagulation activation, but rather an endogenous attempt to counteract impaired vascular and metabolic homeostasis [23]. In contrast, the reduction in talin-1 (TLN1) in obesity/pre-bariatric states points to altered mechanotransduction and cell–matrix interactions. TLN1 is a central focal adhesion protein that links extracellular matrix (ECM) integrins to the intracellular actin and regulates force transmission, adhesion signaling, and extracellular sensing. Interestingly, one study identified TLN1 as a hub gene in type 1 diabetes, and other studies have suggested that TLN1 is involved in glucose intolerance, while its role in adipocytes has yet to be explored [24,25,26].

Our correlation analyses identified several noteworthy associations, providing a more integrated view of how adiposomal signals may track recovery across metabolic, vascular, and inflammatory domains. Neuropilin-1 (NRP1), a transmembrane co-receptor for multiple growth factors (e.g., vascular endothelial growth factor (VEGF), transforming growth factor-beta (TGF-β), hepatocyte growth factor (HGF), and platelet-derived growth factor (PDGF), etc.), showed positive correlations with fasting glucose and HOMA-IR. Research has suggested that NRP1 in adipose macrophages contributes to lipid metabolism and macrophage polarization; loss of NRP1 in macrophages leads to impaired lipid uptake, inflammation, adipose vasculature, and insulin resistance in mouse models [27,28]. However, in humans, the direction of NRP1 changes after weight loss appears to be compartment-dependent: circulating proteomic studies reported increased blood NRP1 after weight loss, whereas adipose tissue-based studies observed decreased NRP1 expression [29,30,31]. In line with the latter findings, our data showed NRP1 decline in adiposomes, suggesting that adiposome-associated NRP1 may reflect adipose-specific metabolic remodeling distinct from that captured by circulating NRP1. Collectively, NRP1 appears to be necessary for adipose tissue homeostasis, yet increased adipose NRP1 may indicate a compensatory response to local metabolic dysfunction rather than preserved tissue health.

Carbonic Anhydrase 1 (CA1) and CA2 were positively associated with total cholesterol and LDL, suggesting that this protein subset may mark a dyslipidemia-linked vascular stress phenotype rather than lipid burden alone. Although our data do not establish causality, this pattern is consistent with evidence linking carbonic anhydrase activity to vascular function, atherosclerotic change, and calcification, as well as with the well-established contribution of LDL to endothelial dysfunction and vascular injury [32,33]. Paraoxonase 3 (PON3), by contrast, was directly associated with fat percentage and visceral fat mass. Consistent with our findings, a previous study reported that serum PON3 significantly decreased after weight loss in overweight/or obese type 2 diabetes participants, while another study demonstrated that PON3 methylation levels could predict weight loss response in obese individuals [33,34]. PON3 is generally regarded as an anti-oxidative, anti-inflammatory, and atheroprotective factor; it can prevent oxidation of LDL in circulation and reduce ROS in mitochondria; therefore, PON3 is essential for metabolic health at both the extracellular and intracellular scenarios. However, serum PON3 has been found to increase in different cardiometabolic diseases, such as peripheral artery disease or coronary artery disease [35]. Hence, the higher expression of PON3, which is, like NRP1, a protective factor in obesity [36], does not necessarily indicate a healthier metabolic state; instead, increased PON3 may represent a compensatory response to adipose dysfunction, oxidative stress, and lipotoxic burden; therefore, its postoperative decline may reflect alleviation of this stress state and progressive restoration of adipose homeostasis.

Collectin subfamily member 11 (COLEC11) and collagen type VI alpha 3 chain (COL6A3) both showed positive correlation with IL-6, while aldolase, fructose-bisphosphate A (ALDOA, a glycolytic enzyme), and complement factor H-related protein 2 (CFHR2, a regulator of the complement system) are related directly to leptin and leptin/adiponectin ratio despite their limited mean-level postoperative change. This pattern is informative because it may suggest that some adiposome proteins may function less as uniformly reversible surgery-responsive signals and more as indicators of residual biological heterogeneity after bariatric surgical intervention. Although these proteins remain underexplored in obesity research, limited but available evidence suggests that they may be involved in obesity-related disorders. For example, COLEC11 has been described as a protective factor against atherosclerosis; the increase in COL6A3 in adipocytes has been associated with adipocyte development and insulin resistance; ALDOA expression in adipose tissue may predict susceptibility to weight gain; and CFHR2 has been observed to be elevated in the circulation of obese females [37,38,39,40]. Collectively, these findings support a model in which bariatric surgery remodels the adiposome proteome in a selective rather than uniform manner: some proteins shift at the group level with surgery, whereas others continue to mirror patient-to-patient differences in residual inflammatory and cardiometabolic risk.

Upstream regulator and pathway analyses provide hypothesis-generating context for how these proteomic changes might relate to functional outcomes. Shifts in adiposome proteomics after bariatric surgery were computationally consistent with reduced activity of key inflammatory regulators in adipose tissue, as IL1B, TNF, IL6, IL17A, and NF-κB were predicted to be inhibited upstream regulators in the IPA; these predictions were not experimentally validated in the present study. Each of these regulators has been implicated in obesity-associated inflammation and insulin resistance. NF-κB is a transcription factor that can be activated in adipocytes under overnutrition stress and has been associated with the production of chemokines and cytokines that recruit macrophages and other immune cells, a process that may perpetuate inflammation and contribute to systemic insulin resistance through mediators such as TNF-α and IL-6 [28,29]. The downregulation of NF-κB signaling after surgery aligns with the reversal of obesity-driven inflammatory activation. With the removal of excess nutrients and fat mass, adipocytes no longer send “distress signals,” breaking the inflammatory loop and reducing chemokine release and immune cell recruitment while allowing existing inflammatory cells to shift toward reparative phenotypes [30].

The canonical pathway analysis identified LXR/RXR and DHCR24, and inhibitor of DNA binding 3 (ID3) as significantly inhibited pathways. ID3, an inhibitor of basic helix–loop–helix (bHLH) transcription factors, is involved in cell differentiation, proliferation, and apoptosis; its upregulation has been observed in various cancers and is especially important for immune escape. Hence, downregulation of the ID3 pathway and neutrophil degranulation may indicate the resolution of acute inflammation and an immune reboot. DHCR24, an essential enzyme in cholesterol biosynthesis, supports lipid metabolism and membrane integrity but is also commonly upregulated in fatty liver and insulin resistance; its reduced activity may reflect a decreased metabolic burden and restored lipid homeostasis. LXR/RXR, although usually considered beneficial for lipid metabolism, is sometimes linked to fatty liver; its downregulation may further indicate alleviated metabolic stress and reduced demand for excessive lipid processing after weight loss.

These changes observed in the FXR/RXR signaling that is known to be involved in the assembly, remodeling, and clearance of plasma proteins align with the observed improvements in lipid metabolism (reduction in atherogenic lipids) and cholesterol trafficking after bariatric surgery, reflecting a more balanced metabolic state. Also, the observed enrichment of insulin signaling (IGF transport and p70S6K signaling) aligns with the improved insulin sensitivity and reduced HOMA-IR observed following surgery. Apart from metabolism, enrichments in pathways related to vascular function were detected as well. For example, atherosclerosis-related signaling and regulation of nitric oxide and reactive oxygen species production in macrophages were achieved in the enrichment analysis, suggesting improved vascular homeostasis. Similarly, pathways related to vascular surface interaction and integrin signaling indicate potential restoration of endothelial integrity and reduced endothelial activation, which is a critical process in early atherogenesis. Finally, the observed enrichment in coagulation pathways, including prothrombin activation and fibrin clot formation, indicates an evident shift from a pro-thrombotic to anti-thrombotic state and reduced cardiovascular risk following bariatric surgery.

Altogether, these findings indicate predicted alterations in inflammatory, proliferative, and lipid-associated pathways following bariatric surgery, consistent with broader shifts in metabolic and immune regulatory networks. Importantly, the integration of these metabolic and vascular pathway changes supports the concept that adiposome remodeling reflects not only improved adipose tissue function but also downstream recovery of endothelial and cardiometabolic health.

This study has several limitations that warrant consideration. First, the relatively small sample size (n = 23) may reduce the generalizability of our findings to broader populations with obesity, particularly to males, other racial/ethnic groups, and individuals with varying degrees of metabolic impairment. Also, a limitation of the current study is that postoperative medications, dietary changes, and other lifestyle-related factors may have influenced the plasma and adipose tissue environment and contributed to the observed adiposome proteomic changes, although the paired longitudinal design helped reduce inter-individual variability. Second, the short follow-up duration of approximately three months post-surgery captures only early postoperative changes and precludes assessment of the long-term sustainability of vascular, metabolic, and proteomic alterations. Third, proteomic analysis was conducted on isolated adiposome fractions, which, while providing mechanistic insight, may not fully represent systemic proteomic changes in plasma or other tissues. Fourth, given the bottom-up proteomics workflow, the identification of intact proteoforms, co-occurring post-translational modifications, and isoform-specific functions was not possible, and the biological roles of some differentially abundant proteins remain incompletely understood. Fifth, certain vascular and metabolic parameters were influenced by potential confounders such as medication changes or dietary adjustments, which were not systematically controlled. Sixth, the protein–clinical correlations reported here are of moderate magnitude (most |r| in the 0.4–0.6 range), and, given the cohort size and the number of comparisons performed, should be regarded as hypothesis-generating signals rather than evidence of strong biological coupling, even after FDR adjustment. Seventh, the artificial neural network analysis was applied to a high-dimensional proteomic feature space relative to the sample size; despite 10-fold cross-validation, the resulting performance estimates remain susceptible to overfitting, and the variable-importance rankings should be interpreted as candidate biomarkers requiring confirmation in independent, adequately powered cohorts before any predictive or clinical use. Additionally, although Top14 high-abundance plasma protein depletion was applied prior to digestion and the lipoprotein marker APOB was undetectable by Western blot in our adiposome preparations, we cannot completely exclude the possibility that a subset of identified proteins, particularly plasma-abundant species such as complement factors (e.g., C3, C5, CFB, CFHR2) and fibrinogens (FGA, FGB, FGG), may, in part, reflect co-isolated soluble proteins rather than exclusively bona fide vesicular cargo; this caveat should be considered when interpreting the absolute composition of the adiposome proteome, especially for proteins that are highly abundant in plasma. Finally, the upstream regulators (including IL6, TNF, and NF-κB) and canonical pathways highlighted by Ingenuity Pathway Analysis are computational predictions inferred from a relatively small set of differentially abundant proteins; these regulators were not validated experimentally in the present study, related pathways may overlap and inflate the apparent breadth of biological interpretation, and the inferences therefore require functional validation in larger, independent cohorts and dedicated mechanistic experiments.

## 4. Materials and Methods

### 4.1. Participants and Ethical Oversight

This investigation was carried out at the University of Illinois Hospital (Chicago, IL, USA). We enrolled 23 adults with obesity (BMI ≥ 30 kg/m^2^) scheduled for sleeve gastrectomy. The study group comprised 12 African American, 9 Hispanic, and 2 non-Hispanic white participants (20 females and 3 males). The mean age was 36.7 years. Fasting blood and physiological measurements were obtained 2–3 weeks prior to surgery. Subcutaneous adipose tissue (SAT) samples were collected intraoperatively and 12–14 weeks after the initial bariatric surgery via biopsy under topical anesthesia (lidocaine) following our published protocols [41]. Exclusion criteria included pregnancy, active tobacco use, prior bariatric surgery, chronic liver or kidney disease, heart failure, cancer, or autoimmune disease. The protocol conformed to the Declaration of Helsinki and was approved by the University of Illinois at Chicago Institutional Review Board (protocol #2021-1113; approval date 25 October 2021). Written informed consent was obtained from all participants before enrollment.

### 4.2. Cardiometabolic Phenotyping

Body weight and BMI were measured in standard fashion. Body composition (percent fat and lean mass) was assessed via dual-energy X-ray absorptiometry using an iDXA scanner (GE Healthcare). Fasting venous samples were analyzed for glucose, insulin, and hemoglobin A1c using our previously validated methods [42]. Insulin resistance was estimated by HOMA-IR: fasting insulin (μU/mL) × fasting glucose (mmol/L) ÷ 22.5 [43]. Lipid panels (total cholesterol, LDL, HDL, triglycerides) were obtained by enzymatic assays (Roche Diagnostics, Indianapolis, IN, USA) following established procedures [42]. Liver chemistries (ALT, AST, bilirubin, albumin, alkaline phosphatase) were measured in the hospital laboratory as part of routine investigations. Plasma nitric oxide bioavailability was indexed by total nitrate/nitrite using a colorimetric kit (Cayman Chemical, Ann Arbor, MI, USA) [44]. Circulating IL-6 and high-sensitivity CRP were quantified by immunoassay (R&D Systems, Minneapolis, MN, USA). Serum adiponectin was measured with a commercial ELISA (Thermo Fisher Scientific; catalog KHP004). Hepatic steatosis was evaluated noninvasively using Attenuation Imaging (ATI) on an Aplio i900 ultrasound platform (Canon Medical Systems, Melville, NY, USA). With a low-frequency curved array transducer, the right hepatic lobe was imaged, and the ATI algorithm produced attenuation coefficients (dB/cm/MHz) from standardized regions of interest as a quantitative marker of liver fat.

### 4.3. Adiposome Isolation and Protein Extraction

WAT biopsies were processed as we previously described [12]. Fresh tissue was rinsed in Medium 199 (Gibco, Waltham, MA, USA), minced, and digested with type I collagenase (Worthington) in Medium 199 containing 4% BSA. The resulting cell suspension was centrifuged at 500× *g* for 1 min to isolate mature adipocytes from the floating layer. The isolated adipocytes were then transferred to membrane inserts and maintained in Medium 199 with 5% exosome-depleted FBS and 1% penicillin-streptomycin. Conditioned medium collected after 24–48 h underwent sequential clearing spins (1000× *g* for 5 min; 15,000× *g* for 15 min), 0.45 µm filtration, and ultracentrifugation at 150,000× *g* for 2 h to pellet adiposomes. Pellets were resuspended and characterized by nanoparticle tracking analysis (NanoSight NS300, Malvern Instruments Ltd., Malvern, Worcestershire, UK). Proteins were extracted from adiposomes with RIPA buffer and quantified using the Pierce BCA assay (Thermo Fisher Scientific, Waltham, MA, USA). Equal protein amounts were resolved on 4–12% Bis-Tris gels, transferred to PVDF, and probed overnight (4 °C) with primary antibodies to CD9, CD81, CD63, PPARγ, adiponectin, APOB, and FABP4 (Cell Signaling Technology, Danvers, MA, USA). IRDye-conjugated secondaries (LI-COR) were used for detection on an Odyssey Clx imager at 700 nm (IRDye680) and 800 nm (IRDye800).

### 4.4. Proteomic Profiling of Adiposome Proteins

Following a brief spin to remove insoluble material, supernatants were quantified by Qubit (values summarized in the accompanying Excel file). To mitigate interference from abundant plasma proteins (e.g., albumin and complement), 300 µg of total protein was incubated with 200 µL Top14 Depletion resin for 15 min, then centrifuged; ~110 µL of depleted supernatant was recovered and re-quantified. For proteomics, 30 µg of depleted protein per sample underwent filter-aided sample preparation (FASP) using 10 kDa MWCO filters. Proteins were reduced with 20 mM DTT (30 min), alkylated with 50 mM IAA in the dark (40 min), and washed with 8 M urea in 0.1 M ammonium bicarbonate (ABC). After equilibration, trypsin digestion proceeded overnight at 37 °C at a 1:30 enzyme:protein ratio.

Peptides were eluted by centrifugation, rinsed with ABC and NaCl, desalted on Oasis PRiME HLB cartridges, dried, and reconstituted in 75 µL of 5% acetonitrile/0.1% formic acid; peptide amounts were verified prior to MS. In total, 46 peptide samples were processed across 17 TMT10-plex sets. Each sample was labeled with TMT10plex reagents following the manufacturer’s protocol, quenched with 4 µL hydroxylamine, pooled, and desalted using Oasis HLB plates. For each pooled set, 200 µg of peptide was fractionated by high-pH reversed-phase chromatography on an XBridge BEH C18 column. Every 11th fraction was concatenated to yield 10 combined fractions, which were dried and reconstituted in 50 µL 5% acetonitrile/0.1% formic acid for LC-MS.

Approximately 1 µg from each pooled fraction was analyzed on a Q Exactive HF mass spectrometer (Thermo Fisher Scientific) coupled to an UltiMate 3000 RSLCnano. Peptides were first trapped and then resolved on Waters nanoEase and BEH C18 columns at 300 nL/min over a 105 min linear gradient (solvent A: 0.1% formic acid in water; solvent B: 0.1% formic acid in 80% acetonitrile). MS1 spectra were acquired at 120,000 resolution over m/z 350–1400. The top 15 precursors (charge 2–5) were fragmented by HCD (NCE = 32%) with a 30 s dynamic exclusion. MS2 scans were collected at 60,000 resolution with AGC targets of 3 × 10^6^ (MS1) and 1 × 10^5^ (MS2) and maximum injection times of 50 ms and 120 ms, respectively. Spectra were searched in Mascot Daemon (v2.6.0) against the UniProt human database (version 11 August 2020) using a 10 ppm parent-ion tolerance. Carbamidomethyl-C was set as a fixed modification; methionine oxidation and N/Q deamidation were variable. Peptide/protein quantification and TMT reporter purity correction were performed in Scaffold DDA (v6.0.1). Identifications required ≥1 confident peptide and were filtered to a 5% FDR at the protein level. To confirm robustness of the identification stringency, the dataset was re-filtered at a stricter 1% protein-level FDR, and the resulting set of differentially abundant proteins was qualitatively unchanged with respect to identity, direction, and rank order of the top hits.

For TMT-based quantification, Scaffold DDA normalization was used to improve comparability across reporter ion channels; total signal normalization was applied by summing TMT reporter ion intensities for each channel and adjusting channel total intensities to equalize total signal across samples. Pooled reference channels were used as internal references to support fold-change calculations and cross-set comparison. In addition, Scaffold also provides a built-in batch correction option to further reduce inter-run variability.

Missing values were systematically cataloged across 333 quantified proteins and 145 paired samples. Of 48,285 total intensity measurements, 2108 (4.37%) were missing, with comparable rates across the three groups (lean: 4.32%, obese: 4.39%, post-surgery: 4.37%); 285 proteins (85.6%) were quantified in all samples, 8 proteins were missing in every sample and were excluded from downstream analyses, and the remaining proteins exhibited intermediate, group-balanced missingness (Supplementary Table S1).

### 4.5. Assessment of Vascular Function

Brachial flow-mediated dilation (FMD) was measured using high-resolution ultrasound (Aplio i900; Canon, Canon Medical Systems Corporation, Otawara, Japan). A cuff placed on the forearm was inflated to 220 mmHg for 5 min. Arterial diameter was recorded 1 min before inflation (baseline) and 5 min after deflation during reactive hyperemia. Images were analyzed with automated edge-detection; FMD (%) was calculated as (peak diameter − baseline diameter)/baseline diameter (peak diameter − baseline diameter)/baseline diameter(peak diameter − baseline diameter)/baseline diameter × 100. For microvascular flow-induced dilation (FID), resistance arterioles were microdissected from adipose tissue, cleared of connective tissue, and mounted on glass microcannulae in a pressurized organ chamber as detailed previously [45]. Vessels were secured with nylon sutures and perfused with Krebs buffer under stepwise intraluminal pressure gradients (10–100 cm H_2_O). Diameters were tracked using an inverted Olympus microscope with video dimension analysis. After preconstriction with endothelin-1 (10^−6^ mol/L), FID was quantified as the percentage increase in diameter at each pressure step relative to the constricted state.

### 4.6. Statistical Analysis and Modeling

Analyses were performed in SPSS v26.0 (SPSS Inc., Chicago, IL, USA) and R 4.4.1 (RStudio). All assays were run in technical triplicate. Continuous data are presented as mean ± SD; categorical variables as counts and percentages. Normality was evaluated by the Shapiro–Wilk. Because pre- and post-surgery measurements were obtained from the same individuals, paired comparisons were performed using the paired Student’s *t*-test for normally distributed variables and the Wilcoxon signed-rank test for non-normally distributed variables. Depending on distributional properties, unpaired comparisons used Student’s *t*-test or ANOVA (with covariate adjustment for age, sex, and race/ethnicity) for parametric data, and the Mann–Whitney U test for non-parametric data. Multiple testing was controlled using the Benjamini–Hochberg FDR, applied separately to differential protein abundance tests and to the protein–clinical correlation matrix; correlation results are reported as nominal Pearson/Spearman coefficients with FDR-adjusted q-values. Log_2_ fold changes were computed as the log_2_ ratio of mean abundance in obese versus lean groups. Associations between protein measures and participant characteristics were examined using linear regression adjusted for age, sex, and race/ethnicity; standardized β coefficients reflect the effect per one-standard deviation (SD) change in predictors. Dietary intake and physical activity levels were assessed using standardized self-reported questionnaires at each study time point. These variables were incorporated into the statistical models to account for potential lifestyle-related confounding effects on clinical and proteomic outcomes.

Principal component analysis (PCA) was conducted on z-scored variables (mean = 0, SD = 1) using prcomp with singular value decomposition. Hierarchical clustering used Ward’s linkage on similarly scaled data, and patterns were visualized via heatmaps. Bar plots, box plots, and volcano plots were generated with ggplot2. Pearson or Spearman correlations were used for linear associations as dictated by normality. Given the modest sample size (n = 23), correlation magnitudes were interpreted using conventional thresholds (|r| < 0.3, weak; 0.3–0.5, moderate; > 0.5, moderate-to-strong) and treated as hypothesis-generating rather than confirmatory; correlations are presented with corresponding 95% confidence intervals where appropriate. Proteins surviving FDR < 0.05 were submitted to Ingenuity Pathway Analysis (IPA); enrichment was determined using Fisher’s exact test. Upstream regulator and canonical pathway predictions generated by IPA are computational inferences based on the abundance and direction of change in input proteins; these predicted regulators (e.g., IL6, TNF, NF-κB) and pathways were not validated experimentally in the present study and should be interpreted as hypothesis-generating.

Artificial neural network (ANN) models were developed using IBM SPSS (version 29) to explore, in a hypothesis-generating manner, candidate adiposome protein predictors of post-surgical cardiometabolic risk factors based on paired adiposome proteomics profiles. Input variables included the quantified concentrations of individual protein species isolated from adiposomes, measured pre- and post-bariatric surgery. Response variables encompassed obesity, type 2 diabetes, hypertension, dyslipidemia, liver steatosis, vascular dysfunction, and elevated systemic inflammation. A multilayer perceptron architecture with one hidden layer was used, employing sigmoid activation functions and trained using the scaled conjugate gradient algorithm. Input features were standardized prior to model fitting. The class imbalance was addressed by assigning inverse-proportional class weights. A 70/30 train-test split was implemented with 10-fold cross-validation to reduce overfitting and ensure model generalizability. Model performance was evaluated based on accuracy, coefficient of determination (R^2^), and mean absolute error (MAE). For each model, the top 10 most informative protein predictors were extracted based on variable importance scores, allowing the identification of key protein species that contributed most significantly to the classification or regression outputs. Feature selection and outcome-specific model parameters were optimized to ensure interpretable outputs. We explicitly acknowledge that, given the small sample size (n = 23) relative to the high feature dimensionality, ANN performance estimates are vulnerable to overfitting even with 10-fold cross-validation. Reported error metrics and variable-importance rankings are therefore reported as exploratory and require external validation in independent, larger cohorts before any predictive or clinical claim can be made.

## 5. Conclusions

Overall, our findings support the view that sleeve gastrectomy induces early recovery across vascular, metabolic, inflammatory, and adipose vesicle domains. The reduction in adiposome abundance, together with coordinated remodeling of inflammatory, immune, redox, and matrix-related cargo, suggests that adiposome proteomic remodeling is associated with local adipose tissue recovery and whole-body cardiometabolic improvement after bariatric surgery and may represent adiposome proteomics as a promising mechanistic layer for future studies of surgical response and postoperative risk stratification.

Future investigations should extend these findings through several complementary approaches. First, larger multicenter cohorts incorporating longitudinal sampling beyond the three-month postoperative period will be essential to differentiate transient postoperative alterations from sustained remodeling of adipose-derived extracellular vesicles. Second, mechanistic studies exposing endothelial cells, macrophages, or hepatocytes to adiposomes isolated before and after surgery could clarify whether the observed shifts in vesicular cargo directly influence nitric oxide signaling, inflammatory activation, lipid metabolism, or extracellular matrix regulation. Such work is particularly important given the growing recognition of adipose-derived extracellular vesicles as key mediators of interorgan metabolic communication in obesity. Finally, targeted validation of candidate proteins, including TLN1, FN1, PRDX2, S100A8, JCHAIN, and adiponectin, would further enhance the biological interpretation and translational relevance of the present dataset.

## Figures and Tables

**Table 1:** Demographics and comorbidities of the participants.

	(n = 23)
Gender (female)	20 (87%)
Race	
African American	12 (52%)
Hispanic	9 (39%)
Non-Hispanic White	2 (9%)
Age (years)	36.7 ± 7.74
Hypertensive	12 (52%)
Diabetic	9 (39%)
Insulin resistant	15 (65%)
Dyslipidemia	15 (65%)
Hepatic steatosis	17 (74%)
Impaired FMD	21 (91%)
Impaired FID	23 (100.0%)
Impaired vascular function	16 (70%)
Systemic inflammation	14 (61%)

FMD, flow-mediated dilation; FID, flow-induced dilation.

**Table 2:** Baseline characteristics of the participants.

	Pre-Surgery (n = 23)	Post-Surgery (n = 23)	Average Change ± SD	*p*-Value
BMI (kg/m^2^)	46.60 ± 6.89	36.90 ± 5.88	−9.70 ± 5.45	<0.01
Heart rate (b/min)	82.65 ± 14.16	73.23 ± 11.80	−9.42 ± 15.22	<0.01
Systolic blood pressure (mmHg)	134.96 ± 20.35	119.48 ± 8.75	−15.48 ± 19.78	<0.01
Diastolic blood pressure (mmHg)	79.13 ± 11.58	76.74 ± 6.02	−2.39 ± 14.93	0.4506
Fasting insulin (µIU/mL)	17.28 ± 7.52	11.42 ± 2.99	−5.86 ± 7.32	<0.01
Fasting glucose (mg/dL)	105.52 ± 23.43	81.30 ± 23.68	−24.22 ± 29.93	<0.01
HOMA-IR	4.80 ± 2.95	2.35 ± 0.98	−2.46 ± 3.14	<0.01
HbA1c (%)	6.35 ± 1.03	5.10 ± 1.31	−1.25 ± 1.52	<0.01
Total cholesterol (mg/dL)	165.39 ± 39.63	131.96 ± 37.24	−33.43 ± 53.92	<0.01
LDL (mg/dL)	89.70 ± 30.14	71.65 ± 25.93	−18.04 ± 39.46	0.0392
HDL (mg/dL)	41.17 ± 10.34	49.91 ± 13.76	+8.74 ± 17.01	0.0220
Triglycerides (mg/dL)	150.57 ± 62.47	112.78 ± 48.50	−37.78 ± 80.88	0.0355
Total bilirubin (mg/dL)	0.43 ± 0.20	0.33 ± 0.16	−0.09 ± 0.27	0.1228
Alkaline phosphatase (U/L)	79.96 ± 15.76	63.96 ± 15.84	−16.00 ± 21.32	<0.01
AST (U/L)	22.39 ± 18.68	18.04 ± 16.36	−4.35 ± 24.81	0.4098
ALT (U/L)	26.35 ± 30.66	21.39 ± 26.52	−4.96 ± 38.87	0.5471
Total protein (g/dL)	7.18 ± 0.36	5.71 ± 0.75	−1.47 ± 0.99	<0.01
Albumin (g/dL)	4.27 ± 0.26	3.40 ± 0.48	−0.87 ± 0.56	<0.01
Hemoglobin (g/dL)	12.04 ± 1.33	14.57 ± 2.23	+2.52 ± 2.21	<0.01

BMI, body mass index; FMD, flow-mediated dilation; FID, flow-induced dilation; HOMA-IR, homeostatic model assessment of insulin resistance; HbA1C, glycated hemoglobin; LDL, low-density lipoprotein; HDL, high-density lipoprotein; AST, aspartate aminotransferase; ALT, alanine aminotransferase; CRP, C-reactive protein; IL6, interleukin-6. Pre- versus post-surgery comparisons were paired; *p*-values were obtained using the paired Student’s *t*-test for normally distributed variables or the Wilcoxon signed-rank test for non-normally distributed variables, as determined by the Shapiro–Wilk test.

**Table 3:** Regulator–target mapping of proteomic abundance.

Target Molecules of Significant Difference	Regulators	Diseases and Functions	Consistency Score	Nodal Total	Known Regulator-Disease/Function Relationship
ADIPOQ, AHSG, ANPEP, CLU, ECM1, FN1, MYH9, NRP1, VTN	BMP10, EGF, F2	Activation of macrophages, migration of mononuclear leukocytes, and migration of smooth muscle cells	7	15	44% (4/9)
ADIPOQ, AGT, ANPEP, APCS, C3, CLU, CRP, FN1, S100A8, SERPINF1, VTN	CEBPA, CEBPB, IL20	Activation of myeloid cells	3.015	15	67% (2/3)
AGT, APOE, CFB	NFKB (complex)	Systolic pressure	1.732	5	0% (0/1)
AGT, APOA1, APOC3, APOE	INSULIN (family), RARA	Hydrolysis of lipid	1.5	7	50% (1/2)
ADIPOQ, AGT, ANPEP, APCS, C3, CLU, CRP, FN1	CEBPB	Inflammatory response	−3.536	10	100% (1/1)
ADIPOQ, AGT, APOE, CLU, FN1, MYH9, NRP1	TNF	Migration of smooth muscle cells	−3.78	9	100% (1/1)
ADIPOQ, AGT, S100A8, SERPINF1	CEBPA	Apoptosis of endothelial cells	−6	6	0% (0/1)
APCS, APOE, C3, C5, CLU, CRP, FGG, FN1, HSPG2, S100A8, SERPINF2, TLN1	IL1B	Activation of blood cells	−6.928	14	100% (1/1)
ADIPOQ, CRP, HPX	CEBPB	Damage to endothelial cells	−7.506	5	0% (0/1)
A2M, AGT, APOE, C3, CRP, FGG, FN1	NFKB (complex)	Secretion of molecules	−9.827	9	100% (1/1)

Adiponectin (ADIPOQ), alpha-2-HS-glycoprotein (AHSG), alanyl aminopeptidase (ANPEP), apolipoprotein C-III (APOC3), apolipoprotein E (APOE), apolipoprotein serum amyloid P component (APCS), apolipoprotein A1 (APOA1), angiotensinogen (AGT), C-reactive protein (CRP), clusterin (CLU), complement component 3 (C3), complement component 5 (C5), complement factor B (CFB), cytochrome C (CYCS), fibronectin 1 (FN1), fibrinogen gamma chain (FGG), glucocorticoid receptor (GCR), heat shock proteoglycan 2 (HSPG2), hemopexin (HPX), myosin heavy chain 9 (MYH9), neuropilin 1 (NRP1), S100 calcium-binding protein A8 (S100A8), serum amyloid A1 (SAA1), serpin family F member 1 (SERPINF1), serpin family A member 3 (SERPINA3), serpin family F member 2 (SERPINF2), talin 1 (TLN1), transthyretin (TTR), Vascular cell adhesion molecule 1 (VCAM1), tumor necrosis factor (TNF), interleukin 1 beta (IL1B), interleukin 20 (IL20), nuclear factor kappa B (NFKB complex), CCAAT/enhancer-binding protein alpha (CEBPA), CCAAT/enhancer-binding protein beta (CEBPB), retinoic acid receptor alpha (RARA), insulin (INSULIN family), bone morphogenetic protein 10 (BMP10), epidermal growth factor (EGF), coagulation factor II/thrombin (F2). Bold molecules are of significant difference between obese and lean participants.

## Data Availability

The mass spectrometry proteomics data generated in this study have been deposited to the ProteomeXchange Consortium via the PRIDE partner repository (https://www.ebi.ac.uk/pride/, accessed on 29 May 2026) under accession number PXD078504. The datasets analyzed during the current study are available from the corresponding author upon reasonable request.

## Supplementary Materials

The following supporting information can be downloaded at: https://www.mdpi.com/article/10.3390/ijms27114939/s1.
