# Supplementary material for: Proteomic Signatures of Adiposomes Track Cardiometabolic Risk Reduction Following Bariatric Surgery
Source: Int J Mol Sci. 2026 May 29;27(11):4939. doi: 10.3390/ijms27114939 (PMC13257330; doi:10.3390/ijms27114939)
Supplement: Supplementary file 1 [file ijms-27-04939-s001.zip › ijms-4260604-supplementary.pdf]

International Journal of
Molecular Sciences

MDPI

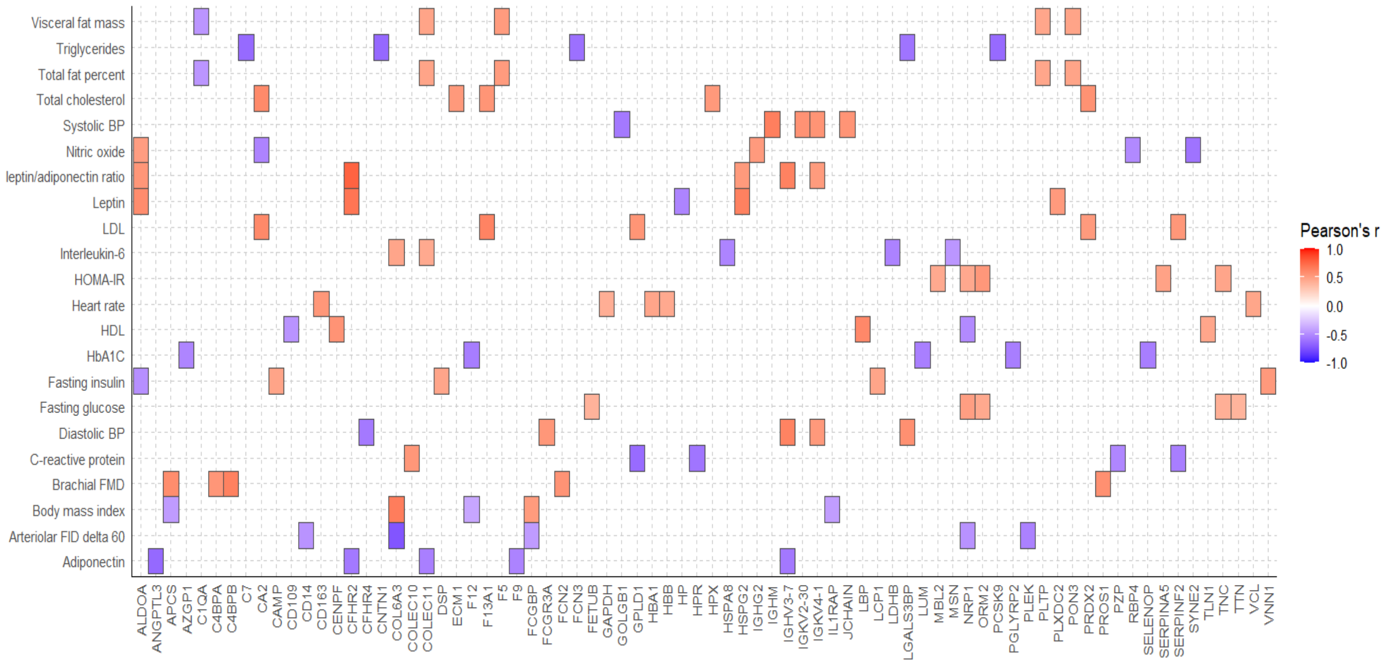

Supplementary Figure S1. Regression analysis and association of adiposome proteins with obesity status. Associations between changes in protein abundance and changes in clinical parameters were assessed using Pearson or Spearman correlation as dictated by Shapiro–Wilk normality testing; reported r values are accompanied by p-values, and given the cohort size (n = 23), correlations are interpreted as hypothesis-generating (|r| < 0.3 weak; 0.3–0.5 moderate; > 0.5 moderate-to-strong).

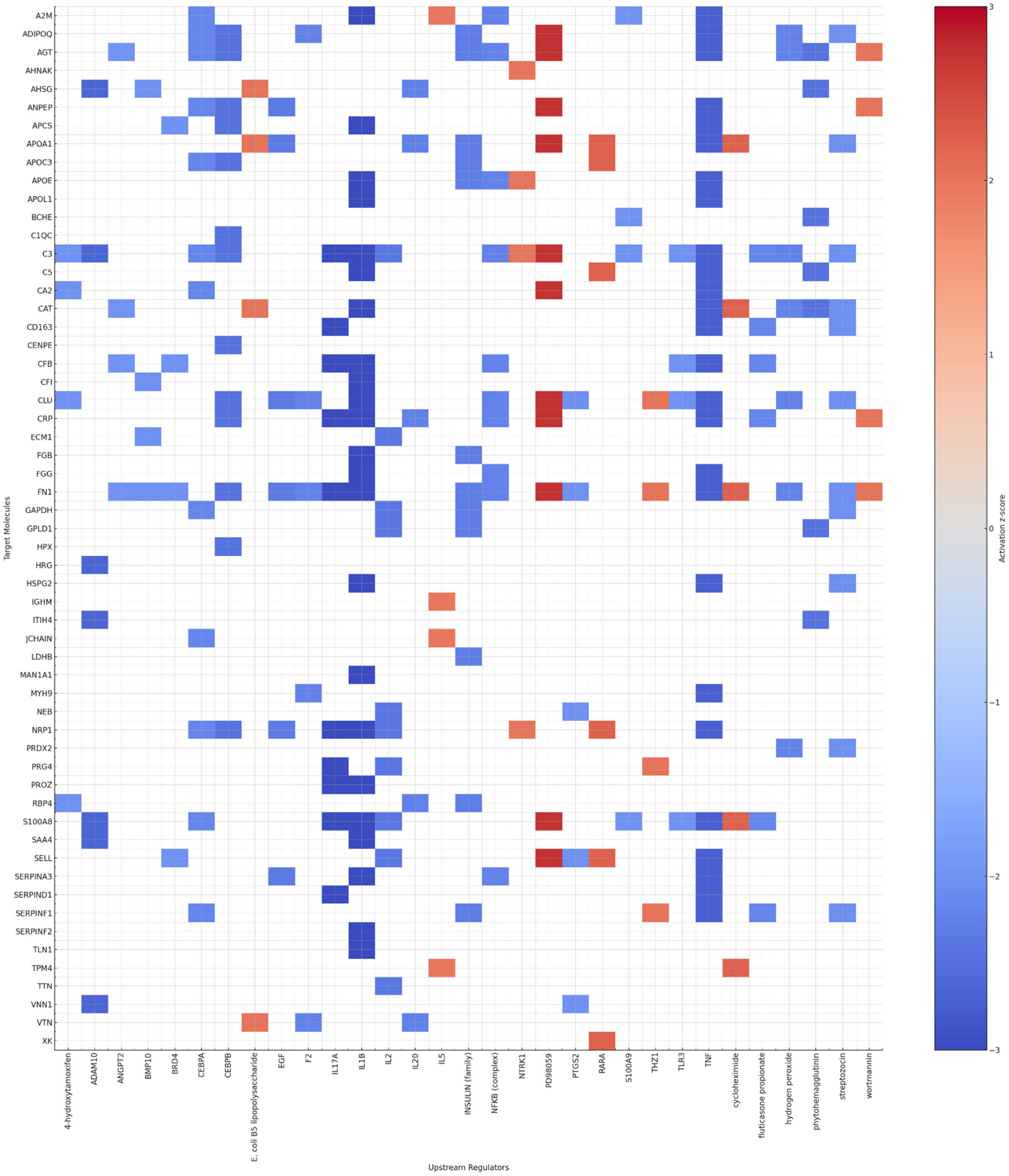

Supplementary Figure S2. Heatmap of z-scores (target molecules vs upstream regulators). Activation z-scores and overlap p-values were computed by Ingenuity Pathway Analysis (Fisher's exact test); regulators shown were filtered by p < 0.05 and |z| > 1.96. These predictions are computational inferences and were not validated experimentally.

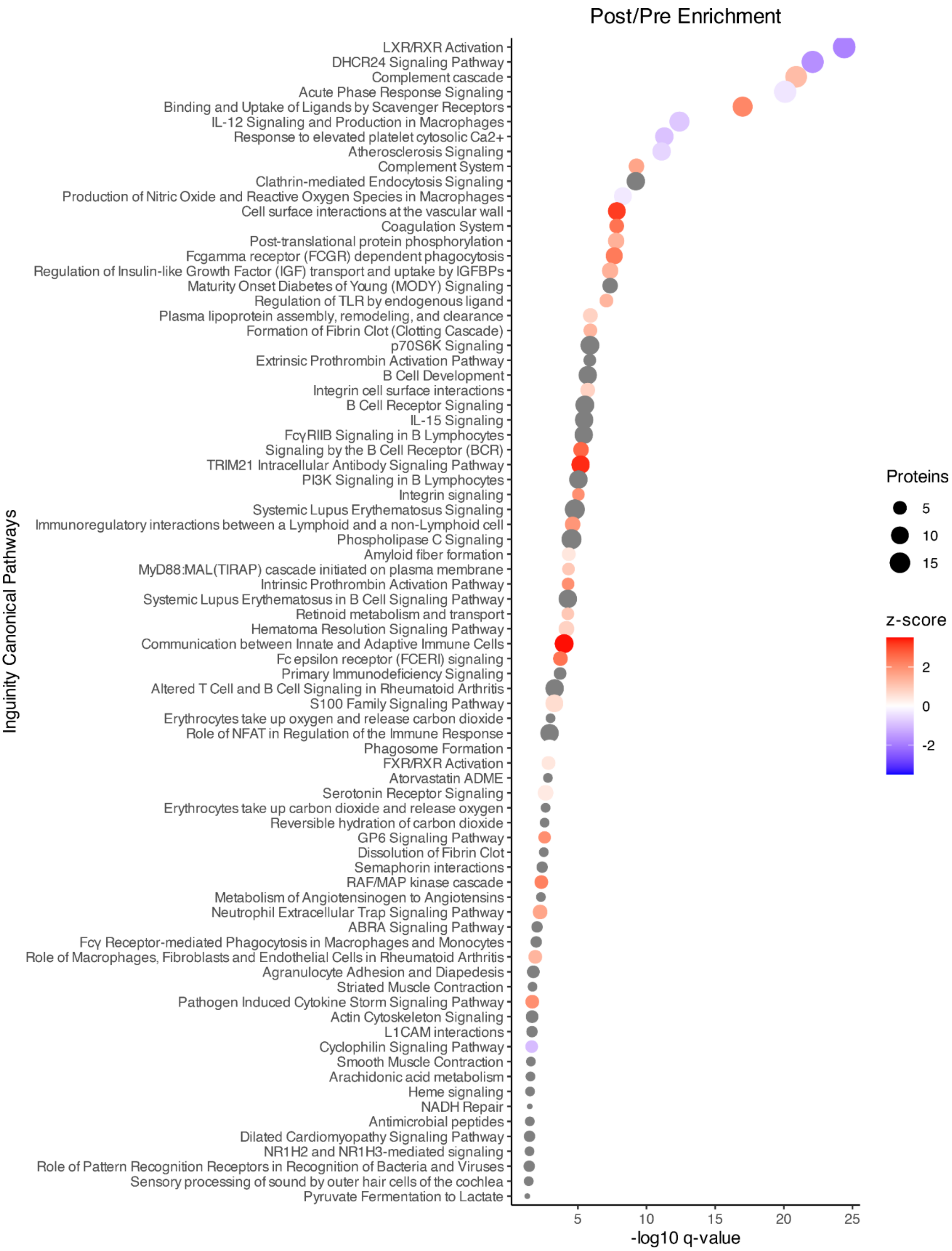

Supplementary Figure S3. Canonical pathways; z-score vs. -log (B-H p-value). Pathway enrichment was computed by Ingenuity Pathway Analysis using Fisher's exact test, with multiple-testing correction by the Benjamini–Hochberg (B-H) procedure;

activation z-scores indicate predicted directional shift. The pathway predictions shown are computational inferences and were not experimentally validated.

**Supplementary Table S1. Missing-value report for the adiposome proteomics dataset.**							
**Overall summary**							
**Metric**	**Value**						
**Total proteins quantified**	333						
**Total samples**	145						
**Total intensity measurements**	48285						
**Missing measurements (n)**	2108						
**Overall missing rate (%)**	4.37						
**Proteins with no missing values**	285						
**Proteins fully missing (excluded)**	8						

**Proteins with intermediate missingness**	40						
**Per-group summary**							
**Group**	**Samples (n)**	**Proteins (n)**	**Total measurements**	**Missing values (n)**	**Missing rate (%)**	**Mean missing/sample**	**Proteins fully missing (group)**
**Lean**	47	333	15651	676	4.32	14.38	8
**Obese**	75	333	24975	1097	4.39	14.63	8
**Post**	23	333	7659	335	4.37	14.57	8
**Per-protein detail (proteins with ≥1 missing measurement)**							
#	**Gene Name**	**Accession**	**Protein Name**	**Missing (n)**	**Missing % (overall)**	**Missing % (Lean)**	**Missing % (Obese)**
1	**IGKV1D-33**	P01593	Immunoglobulin kappa variable 1D-33	145	100.00	100.00	100.00
2	**IGKV1-33**	P01594	Immunoglobulin kappa variable 1-33	145	100.00	100.00	100.00
3	**IGLC3**	P0DOY3	Immunoglobulin lambda constant 3	145	100.00	100.00	100.00
4	**IGLC2**	P0DOY2	Immunoglobulin lambda constant 2	145	100.00	100.00	100.00
5	**IGHV3-30**	P01768	Immunoglobulin heavy variable 3-30	145	100.00	100.00	100.00
6	**IGHV3-30-5**	P0DP03	Immunoglobulin heavy variable 3-30-5	145	100.00	100.00	100.00
7	**UBC**	P0CG48	Polyubiquitin-C	145	100.00	100.00	100.00
8	**UBB**	P0CG47	Polyubiquitin-B	145	100.00	100.00	100.00
9	**KRT31**	Q15323	Keratin, type I	100	68.97	65.96	70.67

			cuticular Ha1				
10	**HBG1**	P69891	Hemoglobin subunit gamma-1	93	64.14	63.83	62.67
11	**HBD**	P02042	Hemoglobin subunit delta	77	53.10	51.06	53.33
12	**CKM**	P06732	Creatine kinase M-type	53	36.55	38.30	38.67
13	**ACTN1**	P12814	Alpha-actinin-1	44	30.35	27.66	30.67
14	**IGLV3-19**	P01714	Immunoglobulin lambda variable 3-19	35	24.14	25.53	25.33
15	**SSC5D**	A1L4H1	Soluble scavenger receptor cysteine-rich domain-containing protein SSC5D	33	22.76	21.28	21.33
16	**CFHR3**	Q02985	Complement factor H-related protein 3	27	18.62	19.15	20.00
17	**NAGLU**	P54802	Alpha-N-acetylglucosaminidase	26	17.93	17.02	18.67
18		Q6Y7W6-DECOY	Q6Y7W6-DECOY	25	17.24	17.02	17.33
19	**LTF**	P02788	Lactotransferrin	25	17.24	14.89	17.33
20	**KRT13**	P13646	Keratin, type I cytoskeletal 13	24	16.55	14.89	16.00
21	**MINPP1**	Q9UNW1	Multiple inositol polyphosphate phosphatase 1	24	16.55	17.02	14.67
22	**KRT17**	Q04695	Keratin, type I cytoskeletal 17	24	16.55	17.02	14.67
23	**KRT14**	P02533	Keratin, type I cytoskeletal 14	21	14.48	12.77	16.00
24	**LYVE1**	Q9Y5Y7	Lymphatic vessel endothelial hyaluronic acid receptor 1	18	12.41	12.77	13.33
25	**BLVRB**	P30043	Flavin reductase (NADPH)	18	12.41	12.77	13.33
26	**CFL1**	P23528	Cofilin-1	18	12.41	12.77	13.33
27	**GDI2**	P50395	Rab GDP	18	12.41	12.77	13.33

https://doi.org/10.3390/xxxxx

			dissociation inhibitor beta				
28	**CAP1**	Q01518	Adenylyl cyclase-associated protein 1	18	12.41	12.77	13.33
29	**PFN1**	P07737	Profilin-1	18	12.41	12.77	13.33
30	**IGF2**	P01344	Insulin-like growth factor II	18	12.41	12.77	13.33
31	**PKM**	P14618	Pyruvate kinase PKM	17	11.72	10.64	12.00
32	**KRT4**	P19013	Keratin, type II cytoskeletal 4	17	11.72	10.64	12.00
33	**SELENBP1**	Q13228	Methanethiol oxidase	17	11.72	10.64	12.00
34	**MMP2**	P08253	72 kDa type IV collagenase	17	11.72	12.77	10.67
35	**S100A9**	P06702	Protein S100-A9	16	11.03	8.51	10.67
36	**KRT16**	P08779	Keratin, type I cytoskeletal 16	9	6.21	6.38	6.67
37		P0DOX8	Immunoglobulin lambda-1 light chain	9	6.21	6.38	6.67
38	**IGHV3-49**	A0A0A0MS15	Immunoglobulin heavy variable 3-49	9	6.21	6.38	6.67
39	**APOC4**	P55056	Apolipoprotein C-IV	9	6.21	6.38	6.67
40	**LRP1**	Q07954	Prolow-density lipoprotein receptor-related protein 1	9	6.21	6.38	6.67
41	**FERMT3**	Q86UX7	Fermitin family homolog 3	9	6.21	6.38	6.67
42	**SAA2**	P0DJI9	Serum amyloid A-2 protein	9	6.21	6.38	6.67
43	**KRT5**	P13647	Keratin, type II cytoskeletal 5	8	5.52	4.26	5.33
44	**HSP90B1**	P14625	Endoplasmin	8	5.52	4.26	5.33
45	**H6PD**	O95479	GDH/6PGL endoplasmic bifunctional protein	8	5.52	6.38	4.00
46	**ALDOB**	P05062	Fructose-bisphosphate	8	5.52	6.38	4.00

			aldolase B				
47	**SOD3**	P08294	Extracellular superoxide dismutase [Cu-Zn]	8	5.52	4.26	5.33
48	**CHL1**	O00533	Neural cell adhesion molecule L1-like protein	4	2.76	2.13	4.00

https://doi.org/10.3390/xxxxx
